# RNA-Dependent RNA Polymerases of Picornaviruses: From the Structure to Regulatory Mechanisms

**DOI:** 10.3390/v7082829

**Published:** 2015-08-06

**Authors:** Cristina Ferrer-Orta, Diego Ferrero, Núria Verdaguer

**Affiliations:** Molecular Biology Institute of Barcelona (CSIC), Barcelona Science Park (PCB), Baldiri i Reixac 10, Barcelona E-08028, Spain; E-Mails: cfocri@ibmb.csic.es (C.F.-O.); dfecri@ibmb.csic.es (D.F.)

**Keywords:** viral replication, RNA-dependent RNA polymerase, positive-strand RNA viruses, picornaviruses, replication fidelity

## Abstract

RNA viruses typically encode their own RNA-dependent RNA polymerase (RdRP) to ensure genome replication within the infected cells. RdRP function is critical not only for the virus life cycle but also for its adaptive potential. The combination of low fidelity of replication and the absence of proofreading and excision activities within the RdRPs result in high mutation frequencies that allow these viruses a rapid adaptation to changing environments. In this review, we summarize the current knowledge about structural and functional aspects on RdRP catalytic complexes, focused mainly in the *Picornaviridae* family. The structural data currently available from these viruses provided high-resolution snapshots for a range of conformational states associated to RNA template-primer binding, rNTP recognition, catalysis and chain translocation. As these enzymes are major targets for the development of antiviral compounds, such structural information is essential for the design of new therapies.

## 1. Introduction

**Figure 1:**
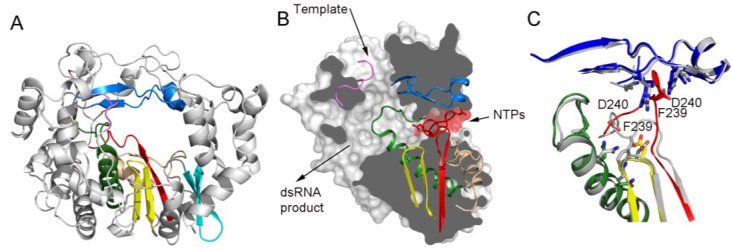

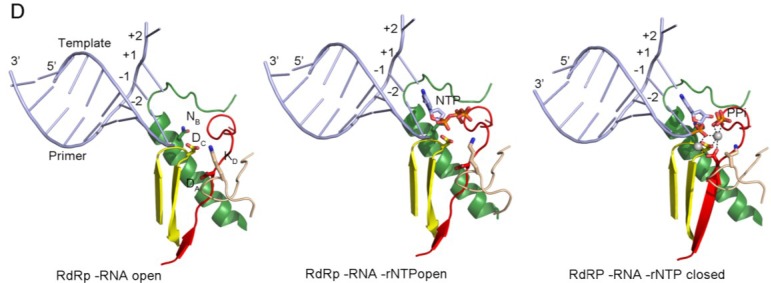
Overall structure of a viral RdRP. (**A**) Ribbon representation of a typical picornaviral RdRP (model from the cardiovirus EMCV 3D^pol^, PDB id. 4NZ0). The seven conserved motifs are indicated in different colours: motif A, red; motif B, green; motif C, yellow; motif D, sand; motif E, cyan; motif F, blue; motif G, pink; (**B**) Lateral view of a surface representation of the enzyme (grey) that has been cut to expose the three channels that are the entry and exit sites of the different substrates and reaction products. The structural elements that support motifs A–G are also shown as ribbons. This panel also shows the organization of the palm sub-domain with motif A shown in two alternative conformations: the standard conformation (PDB id. 4NZ0) found in the apo-form of most crystallized 3D^pol^ proteins and the altered conformation found int the tetragonal crystal form of the EMCV enzyme (PDB id. 4NYZ). The alterations affect mainly Asp240, the amino acid in charge of incoming ribonucleotide triphosphate (rNTP) selection, and the neighboring Phe239 that move ~10 Å away from its position in the enzyme catalytic cavity directed towards the entrance of the nucleotide channel, approaching to motif F; (**C**) Close up of the structural superimposition of the two alternative conformations of the EMCV motif A; (**D**) The PV replication-elongation complexes. Sequential structures illustrating the movement of the different palm residues from a binary PV 3D^pol^-RNA open complex (left) to an open 3D^pol^-RNA-rNTP ternary complex (middle) where the incoming rNTP is positioned in the active site for catalysis and, a closed ternary complex (right) after nucleotide incorporation and pyrophosphate (PPi) release. The residues D_A_ (involved in rNTP selection through an interaction with the 2′ hydroxyl group), D_C_ (the catalytic aspartate of motif C), K_D_ (the general acid residue of motif D that can coordinate the export of the PPi group) and N_B_ (a conserved Asn of motif B, interacting with D_A_) have been highlighted as sticks. The different structures correspond to the 3D^pol^-RNA (PDB id. 3OL6), 3D^pol^-RNA-CTP open complex (PDB id. 3OLB) and 3D^pol^-RNA-CTP closed complex (PDB id. 3OL7) structures of PV elongation complexes, respectively [7].

RNA dependent RNA polymerases (RdRPs) are the catalytic components of the RNA replication and transcription machineries and the central players in the life cycle of RNA viruses. RdRPs belong to the superfamily of template-directed nucleic acid polymerases, including DNA-dependent DNA polymerases (DdDP), DNA-dependent RNA polymerases and Reverse Transcriptases (RT). All these enzymes share a cupped right hand structure, including fingers, palms and thumb domains, and catalyze phosphodiester bond formation through a conserved two-metal ion mechanism [1]. A structural feature unique to RdRPs is the “closed-hand” conformation, in opposition to the “open-hand” found in other polynucleotide polymerases. This “close-hand” conformation is accomplished by interconnecting the finger and thumb domains through the N-terminal portion of the protein and several loops protruding from fingers, named the fingertips that completely encircle the active site of the enzyme [2,3]. In the prototypic RdRPs the closed “right hand” architecture encircles seven motifs (A to G) conserved in sequence and structure (Figure 1), playing critical roles in substrate recognition and catalysis. Three well-defined channels have been identified in the RdRP structures, serving as: the entry path for template (template channel) and for nucleoside triphosphates (NTP channel) and the exit path for the dsRNA product (central channel) (Figure 1B).

The *Picornaviridae* family is one of the largest virus families known, including many important human and animal pathogens. Picornaviruses are non-enveloped RNA viruses possessing a single-stranded RNA genome (7–8 kb) of positive polarity, with a small peptide (VPg; from 19 to 26 amino acids long) linked to its 5′-end. Their genomes have a long highly structured 5′ nontranslated region (NTR), a single large open reading frame (ORF) and a short 3′ NTR, terminated with a poly(A) tail. The ORF is translated in the cytoplasm of the host cell into a polyprotein, which is proteolytically processed by viral proteases to release the structural proteins (VP1-4), needed to assemble virus capsids and the nonstructural proteins (2A-2B-2C-3A-3B-3C^pro^-3D^pol^ and in some genera L) as well as some stable precursors necessary for virus replication in host cells [4]. The picornavirus genome is replicated via a negative-sense RNA intermediate by the viral RdRP, named 3D^pol^. This enzyme uses VPg (the product of 3B) as a primer to initiate the replication process. The structure and function of 3D^pol^ has been studied extensively in the past decades and, to date, the 3D^pol^ crystal structures have been reported for six different members of the enterovirus genus [poliovirus (PV), coxsackievirus B3 (CVB3), enterovirus 71 (EV71) and the human rhinoviruses HRV1B, HRV14, and HRV16], for the aphthovirus FMDV and for the cardiovirus EMCV, either isolated or bound to different substrates [5,6,7,8,9,10,11,12,13,14,15]. These structures provided insights into both initiation of RNA synthesis and the replication elongation processes. Furthermore, mutational analyses in PV and FMDV also have demonstrated that some substitutions in residues located far from the active site, in particular at the polymerase N terminus, have significant effects on catalysis and fidelity. All of these observations suggest that nucleotide binding and incorporation are modulated by a long-distance network of interactions [5,16,17,18,19,20,21,22].

## 2. VPg Binding to 3D^pol^ and Initiation of RNA Synthesis

Correct initiation of RNA synthesis is essential for the integrity of the viral genome. There are two main mechanisms by which viral replication can be initiated: primer-independent or *de novo*, and primer-dependent initiation, reviewed in [23]. Briefly, in the *de novo* synthesis, one initiation nucleotide provides the 3′-hydroxyl for the addition of the next nucleotide whereas the primer dependent initiation requires the use of either an oligonucleotide or a protein primer as provider of the hydroxyl nucleophile. It is remarkable that the RdRPs of viruses that initiate replication using *de novo* mechanisms (*i.e.*, members of the *Flaviviridae* family) share a number of unique features which ensure efficient and accurate initiation, including a larger thumb subdomain containing structural elements that fill most of the active site cavity, providing a support platform for the primer nucleotides (reviewed in [24,25]). These protrusions also serve as a physical barrier preventing chain elongation. Therefore, it is necessary that the initiation platform can move away from the active site after stabilizing the initiation complex, allowing the transition from initiation to elongation [26,27,28]. By contrast, the members of the *Picornaviridae* and *Caliciviridae* families use exclusively the protein-primed mechanism of initiation. The RNA polymerases of these viruses use VPg as primer for both minus and plus strand RNA synthesis. These enzymes display a more accessible active site cavity, enabling them to accommodate the primer protein for RNA synthesis [13,29].

The very first step in protein-primed initiation in picornavirus is the uridylylation of a strictly conserved tyrosine residue of VPg [30]. In this process, the viral polymerase 3D catalyzes the binding of two uridine monophosphate (UMP) molecules to the hydroxyl group of this tyrosine using as template a cis-replicating element (cre) that is located at different positions of the RNA genome, in the different picornaviridae genera (see [31] for an extensive review). The nucleotidylylation reaction can, however, also occur in a template-independent manner in other viruses, for example in caliciviruses [32].

The picornaviral proteins VPg, 3D^pol^ and 3C^pro^, alone or in the 3CD precursor form, together with the viral RNA *cre* elements comprise the so-called “VPg uridylylation complex” responsible for VPg uridylylation *in vivo*. Despite extensive structural and biochemical studies, there are several different models for the interactions established between VPg and 3D^pol^ or 3CD in the uridylylation complex and the precise mechanism of uridylylation remains uncertain [31].

**Figure 2:**
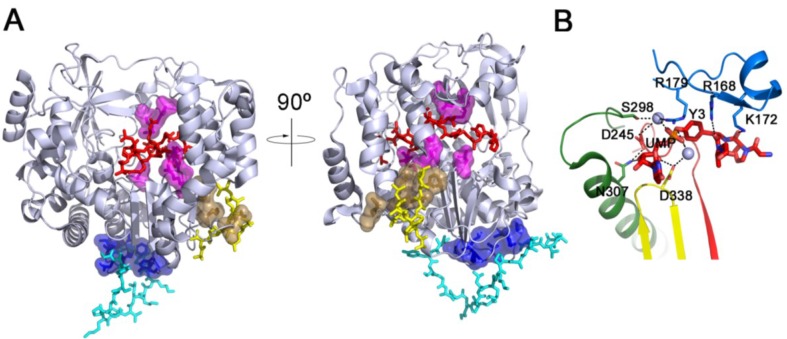
(**A**) Comparison of identified VPg binding sites in picornavirus 3D^pols^. Because all reported structures of picornavirus 3D^pols^ share high structural similarities, we used the structure of the FMDV 3D^pol^ (PDB id. 2F8E, [2]) as a representative model in this figure and colored it with a light-blue cartoon. The bound VPgs with FMDV [13], CVB3 (PDB id. 3CDW, [8]) and EV71 (PDB id. IKA4, [34]) are shown as red, yellow and cyan sticks, respectively. The residues for VPg (or 3AB) binding in PV (F377, R379, E382 and V391) [33], FMDV (E166, R179, D338, D387 and R388) [13] and EV71 (T313, F314, I317, L319, D320, Y335 and P337) [34] 3D^pol^ are represented as a surface and colored as sand, magenta and blue, respectively, in the cartoon representation; (**B**) Details of the interactions described in the active site of the FMDV 3D^pol^ during the uridylylation reaction. The VPg residues and UMP covalently linked are shown in red sticks, the divalent cations are shown as light-blue spheres and the amino acids involved in uridylylation reaction are shown as sticks. The motifs A, B, C and F are colored in red, green, yellow and blue, respectively.

Biochemical and structural studies performed for different members of the family: PV [33], HRV16 [12], FMDV [13], CVB3 [8] and EV71 [34] revealed three distinct VPg binding sites on 3D^pol^ (Figure 2). Strikingly, whereas most picornaviruses express only a single VPg protein, FMDV possesses three similar but not identical copies of VPg: VPg1, VPg2 and VPg3 [35], all of which are found linked to viral RNA [36]. Although not all the copies are needed to maintain infectivity [37,38], there are no reports of naturally occurring FMDV strains with fewer than three copies of 3B, suggesting that there is a strong selective pressure towards maintaining this redundancy [39,40].

The structure of two complexes between FMDV 3D^pol^ and VPg1: 3D^pol^-VPg1 and 3D^pol^-VPg1-UMP revealed a number of residues in the active site cleft of the polymerase involved in VPg binding and in the uridylylation reaction. Functional assays performed with 3D^pol^ and VPg mutants with substitutions in residues involved in interactions, according to the structural data, showed important effects in uridylylation [13]. The position of VPg in complex with the FMDV 3D^pol^ is remarkably similar to the position of the primer and RNA duplex product found in the complex with the same enzyme [14,15]. Most of the amino acids of 3D^pol^ seen in contact with the RNA primer and duplex product are also involved in interactions with VPg. In fact, the structure shows how the VPg protein accesses the active site cavity from the front of the molecule through the large RNA binding cleft mimicking, at least in part, the RNA molecule. The N-terminal position of VPg projects into the active site where the hydroxyl moiety of the residue Tyr3 is in good proximity to the catalytic aspartates of motifs A and C (Figure 2). In this position, Tyr3 essentially mimics the 3' OH of the primer strand during the RNA elongation. Conserved residues in the fingers, palm and thumb domains of the polymerase were identified as being responsible for stabilizing VPg in its binding cavity. In the 3D^pol^-VPg1-UMP complex, the hydroxyl group of Tyr3 side chain was found covalently attached to the α-phosphate moiety of the uridine-monophosphate (UMP) molecule [13]. The positively charged residues of motif F also participate in the uridylylation process, stabilizing Tyr3 and UMP in a proper conformation for the reaction [13] (Figure 2B). Two divalent cations, together with the catalytic aspartic acid residues of motifs A and C, participate in VPg uridylylation. All the observed structural features suggest a conservation of the catalytic mechanism described for all polymerases [1]. Mutational analyses at the conserved FMDV 3D^pol^ residues that strongly interact with VPg in the crystal structures show a drastic defect in VPg uridylylation [13]. This “front-loading” model for VPg binding, compatible with a *cis* mechanism of VPg uridylylation was further supported by the crystal structures of HRV16 3D^pol^ [12] and of the PV 3CD precursor [20]. In the latter structure, the extensive crystal packing contacts found between symmetry-related 3CD molecules and the proximity of the N-terminal domain of 3C to the VPg binding site, in the way that VPg was positioned in the FMDV 3D-VPg complex, suggests a possible role of the contacting interfaces in forming and regulating the VPg uridylylation complex during the initiation of viral replication [20].

After nucleotidylylation of VPg, some structural rearrangements of the 3D^pol^ will follow, marking the transition from initiation to the elongation phase of RNA synthesis. There is experimental evidence supporting possible structural differences in 3D^pol^ when involved in the priming reaction *vs.* elongation of RNA. *i.e.*, the nucleoside analog 5-Fluorouridine triphosphate (FUTP) is a potent inhibitor of VPg uridylylation but not of RNA elongation [19]. Furthermore, for the poliovirus, it has been suggested, based on the structure of the FMDV 3D^pol^-VPg complex that a conserved Asn in the polymerase motif B (Asn297) (equivalent to Asn307 in FMDV) interacts with the 3'-OH of the incoming nucleotide in the uridylylation complex, but with the 2'-OH in the elongation complex [41]. The interaction of Asn307 with the incoming rNTP 2' OH during RNA elongation has been confirmed in a number of picornavirus elongation complexes [6,7,15].

A second binding site for VPg was found in the structure of the CVB3 polymerase [8]. The VPg fragment solved, corresponding to the C-terminal half of the peptide, was bound at the base of the thumb sub-domain in an orientation that did not allow its uridylylation by its own carrier 3D^pol^ (Figure 2A). This VPg binding, partially agreed with previous data reported for PV, showing that a number of amino acids located in motif E of poliovirus (PV) were required for VPg or their precursor, 3AB, binding and affecting VPg uridylylation [33]. In light of these results, the authors proposed that VPg bound at this position was either uridylylated by another 3D molecule or that it played a stabilizing role within the uridylylation complex [8].

Finally, a third VPg binding site was discovered in the structure of the EV71 3D^pol^-VPg complex. In this complex, VPg is anchored at the bottom of the palm domain of the polymerase, showing a V-shape conformation that crosses from the front side of the catalytic site to the back side of the enzyme (Figure 2A). Similarly to that occurring in the previously studied viruses, the mutational analyses of the interacting residues evidenced a reduced binding of VPg to the EV71 3D^pol^ affecting uridylylation [34,42]. Additional experiments performed by the same authors, by mixing the VPg-binding-defective mutants with catalytic defective mutant of the EV71 polymerase, demonstrated *trans* complementation of VPg uridylylation *in vitro*. However, the structure of the EV71 3D^pol^-VPg complex showed that the VPg Tyr3 is buried at the base of the polymerase palm indicating that a conformational change should occur to expose the side chain of Tyr3 for uridylylation.

Taking into account the important sequence homology between the picornaviral VPg sequences [13,43] and the high similarities existing in the 3D^pol^ structures, it is tempting to speculate that the three different VPg binding sites observed in the different crystal structures might reflect distinct binding positions of VPg to both 3D^pol^ or its precursor 3CD at different stages of the virus replication initiation process. As discussed above, the FMDV genome codes for three VPg molecules, all of them are present in naturally occurring viruses [39,40]. A global picture of the assembly of the multicomponent complexes involved in replication initiation in Picornaviruses and its regulation would require the structural and functional analyses of higher order complexes formed by the polymerase 3D^pol^, involving different proteins or protein precursors (VPg, 3AB, 3C^pro^, 3CD) and RNA templates. Such structures would shed new light on the molecular events underlying the initiation of RNA genome replication in these viruses, and should provide crucial information for the design of new antiviral strategies.

## 3. Structural Elements Regulating Replication Elongation in RdRPs

### 3.1. Subtle Conformational Changes Associated with Nucleotide Selection and Active Site Closure for Catalysis

The replication elongation process can be roughly divided in three steps, including nucleotide selection, phosphodiester bond formation and translocation to the next nucleotide for the subsequent round of nucleotide addition. Structural biology has been crucial to elucidate the structural changes associated with each phase of catalysis for a wide number of polymerases [25,29,44,45]. Extensive biochemical and structural studies in the A- and B-families of open-hand nucleic acid polymerases indicate that the movement of an α-helix of the fingers sub-domain would control each step of the nucleotide-addition cycle and facilitates translocation along the template after catalysis [46,47]. In contrast in the “closed-hand” RdRPs, the presence of the fingertips encircling the catalytic site constrains the fingers’ movement relative to the thumb, avoiding the swinging movement of the fingers that is associated with active site closure in open-hand polymerases.

The structures of a large number of RdRP-RNA-rNTP replication-elongation complexes determined, for different members of both, the *Picornaviridae* and the *Caliciviridae* families, have provided important insights into the structural changes associated to each catalytic step [3,6,7,25,29,48,49,50,51]. These structures indicate that RdRPs use subtle rearrangements within the palm domain to fully structure the active site for catalysis upon correct rNTP binding. Briefly, in a first state, the RdRP-RNA complex in the absence of an incoming rNTP, shows an open conformation of the polymerase active site characterized by a partially formed three-stranded β-sheet of the palm domain motifs A and C (Figure 1B,C). A second feature characterizing this state is the presence of a fully prepositioned templating nucleotide (t+1), sitting above the active site and stacked on the upstream duplex and ready for the binding of the incoming rNTP (Figure 1C). In a second state, an incoming rNTP reaches the active site and establishes base-pairing interactions with the template base (t+1), but catalysis has not taken place because the catalytic site is still in open conformation. The third state occurs after binding of the correct nucleotide to the active site. This binding induces the realignment of β-strands in the palm subdomain that includes the structural motifs A and C, resulting in the repositioning of the motif A aspartate to allow interactions with both metal ions required for catalysis [6,7,48,49]. It is important to remark that the active site closure in RdRPs is triggered by the correct nucleotide binding, suggesting that nucleotide selection in RdRPs is a simple process in which base-pairing interactions control the initial rNTP binding geometry and the resulting positioning of the ribose hydroxyls becomes the major checkpoint for proper incoming nucleotide selection. In particular, two residues within motif B (Ser and Asn) and, a second Asp residue at the C-terminus of motif A, strictly conserved among picorna- and caliciviruses form the ribose binding pocket (Figure 1C). The interactions between these amino acids and the ribose hydroxyl groups of the incoming rNTP would stabilize the subtle restructuring of the palm domain that results in the formation of a functional active site. An incorrect nucleotide can bind, but its ribose hydroxyls will not be correctly positioned for active site closure, in consequence, the incorporation efficiency will be reduced.

In addition, growing amounts of data indicate that the conformational changes in motif D determine both efficiency and fidelity of nucleotide addition [52,53]. Biochemical studies of nucleotidyl transfer reactions catalyzed by RdRPs, RTs and single subunit DNA polymerases made the unexpected observation that two protons, not just one, are transferred during the reaction and that the second proton derives from a basic amino acid of the polymerase (termed general acid) and is transferred to the PPi leaving group [52]. PPi protonation is not essential but contributes from 50-fold to 1000-fold the rate of nucleotide addition. Additional data from mutagenesis and kinetics of nucleotide incorporation showed that the general acid was a lysine located in the conserved motif D of RdRPs and RTs [53]. Solution NMR studies were used to analyze the changes that occurred during nucleotide addition. A methionine within motif D, located in the vicinity of the conserved lysine, was found to be a very informative probe for the positioning of the motif. Authors have found that the constitution of the catalytically competent elongation complex (RdRP-RNA-NTP) required the formation of a hydrogen bond between the β-phosphate of incoming rNTP and motif D lysine [54]. The protonation state of this lysine was also observed to be critical to achieve the closed conformation of the active site. Moreover, the ability of motif D to reach the catalytically competent conformation seems to be hindered by the binding of an incorrect nucleotide and this ability continues to be affected after nucleotide misincorporation. Indeed, the NMR data correlates the conformational dynamics of motif D to the efficiency and fidelity of nucleotide incorporation [54].

A number of structures of enterovirus elongation complexes have been trapped in a post-catalysis state in which the newly incorporated nucleotide and the pyrophosphate product are still in the active site, and the active site opens its conformation [6,7]. This state will be followed by the translocation of the polymerase by one base pair to position the next templating nucleotide in the active site for the next round of nucleotide addition. A number of crystal structures have also been solved in a post-translocation state [6,7,15]; however, no structures of translocation intermediates are currently available for RdRPs and the precise mechanism is not yet known. Recent data also suggest that a conserved lysine residue within motif D can coordinate the export of the PPi group from the active site once catalysis has taken place [16], thereby triggering the end of the reaction cycle and allowing enzyme translocation. Very recently, structural and functional data in enteroviruses indicate that steric clashes between the motif-B loop and the template RNA would also promote translocation [51] (see below).

Viral RdRPs are considered to be low fidelity enzymes, generating mutations that allow the rapid adaptation of these viruses to different tissue types and host cells. Based on X-ray data of CVB3 and PV catalytic complexes, the laboratories of Peersen and Vignuzzi engineered different point mutations in these viral polymerases and studied their effects on *in vitro* nucleotide discrimination as well as virus growth and genome replication fidelity. Data obtained revealed that the palm mutations produced the greatest effects on *in vitro* nucleotide discrimination and that these effects appeared strongly correlated with elongation rates and *in vivo* mutation frequencies, with faster polymerases having lower fidelity. These findings suggested that picornaviral polymerases have retained a unique palm domain-based active-site closure as a mechanism for the evolutionary fine-tuning replication fidelity and provide a pathway for developing live attenuated virus vaccines based on engineering the polymerase to reduce virus fitness [55,56].

Finally, recent structural data on calicivirus RdRPs have provided evidence of new conformational changes occurring during catalysis. Structural comparison of the human Norovirus (NV) RdRP determined in multiple crystal forms, in the presence and absence of divalent metal cations, nucleoside triphosphates, inhibitors and primer-template duplex RNAs, revealed that in addition to the active site closure, the NV RdRP exhibits two additional key changes: a rotation of the central helix in the thumb domain by 22°, resulting in the formation of a binding pocket for the primer RNA strand and the displacement of the C-terminal tail region away from the central active-site groove, which also allows the rotation of the thumb helix [57].

### 3.2. Conformational Plasticity of the Motif B Loop Regulates RdRP Activity

**Figure 3:**
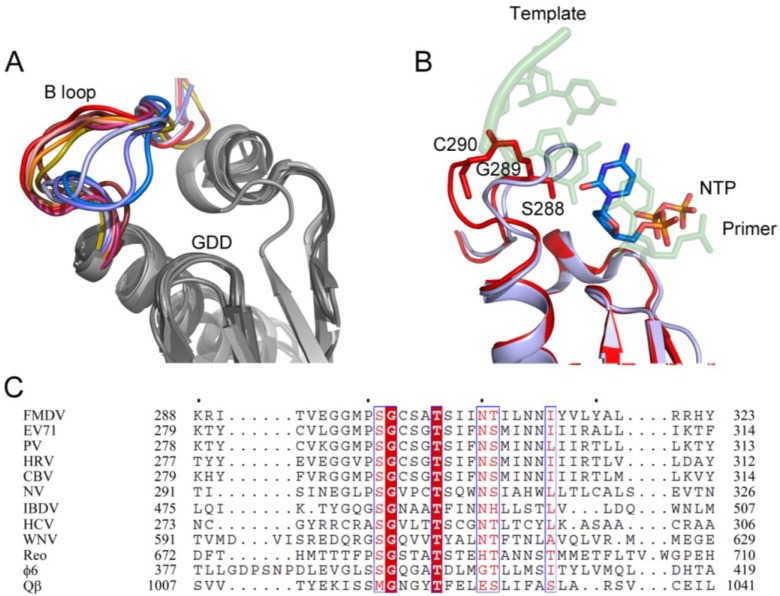
The conformational changes in the B-loop of RdRPs. (**A**) Superposition of the different conformations described for the B-loop. Motifs A, B and C are represented as ribbons and colored in gray tones. The B-loop is shown in different colors for each observed conformation, from red (up) to blue (down): NV NS7, Mg^2+^ bound (PDB id. 1SH3, chain A) chocolate; PV apo-form (PDB id, 1RA6) red; FMDV-RNA complex (PDB id, 1WNE) magenta; PV C290V mutant (PDB id. 4NLP) light-orange; IBDV VP1 + VP3 C-ter peptide (PDB id. 2R70) orange; NV NS7, Mg^2+^ bound (PDB id. 1SH3, chain B) yellow; PV C290F mutant (PDB id. 4NLQ) light-blue; IBDV VP1 apoform (PDB id. 2PUS) slate; FMDV K18E mutant (PDB id. 4WYL) blue; (**B**) Superimposition of the up conformation of PV apo-form (PDB id. 1RA6) red and the down conformation of PV C290F mutant (PDB id. 4NLQ) slate with the RNA template-primer and an incoming rNTP molecule are represented as sticks in semi-transparent representation; (**C**) Sequence alignment of the B-loop region of all the RdRPs from dsRNA and +ssRNA.

The central role of the motif B loop in template binding, incoming nucleotide recognition and correct positioning of the sugar in the ribose-binding pocket was evidenced in the first structures of the FMDV catalytic complexes [15,29]. This loop, connecting the base of the middle finger to the α-helix of motif B, is able to adopt different conformations when it binds to different template and incoming nucleotides, being one the most flexible elements of the active site of RdRPs in picornaviruses, as well as in other viral families (reviewed in [50]). In fact, structural comparisons evidenced large movements of the B-loop, ranging from a conformation in which the loop is packed against the fingers domain leaving the active site cavity fully accessible for template entry, to a configuration where the loop protrudes towards the catalytic cavity and clashes with the template RNA (Figure 3). The key residue of this flexible region is a strictly conserved glycine, which acts as a hinge for the movement. The critical role of the B-loop dynamics was previously anticipated by site-directed mutagenesis in the picornavirus EMCV. Substitutions of the hinge glycine in 3D^pol^ essentially abolished RNA synthesis *in vitro* [58]. Furthermore, additional interactions established between the B-loop and the RNA phosphodiester backbone of the upstream duplex, between the −1 and −2 nucleotides, prompted researchers to hypothesize a function of the loop in modulating polymerase activity through effects on translocation [6,7,15].

Extensive structural and functional work in PV, using several polymerase mutants, harboring substitutions within the B-loop sequence Ser288-Gly289-Cys290, evidenced a major role of these residues in the 3D^pol^ catalytic cycle [51]. The work concluded that the B-loop is able to adopt mainly three major conformations, termed *in*/*up*, *in*/*down* and *out*/*down* and that each alternative conformation is important for the correct NTP binding and for the post-catalysis translocation step. The terms *in*/*out* refer to the loop conformations, packed against the fingers (in), or protruding into the catalytic cavity (out) (Figure 3A). The designation is based on whether the residue Cys290 is buried “in” a hydrophobic pocket directly behind the loop or is “out” of the pocket and exposed to solvent [59]. Moreover, the Sholders and Peersen work highlighted the role of the PV Ser288. The side chain of this residue may also adopt two alternative conformations: pointing “up” toward the ring finger and away from the active site, or “down” pointing toward the active site. These authors propose a sequential model for the structural changes occurred during the PV RdRP catalytic cycle where initially, in the apo-form structure of PV 3D^pol^, the B-loop is in the *in*/*up* conformation, allowing rNTP entry. Equivalent conformations of the B-loop were observed in the apo-forms of 3D^pol^ in FMDV [14], Rhinovirus [11] and Coxsackievirus [8,9]. On nucleotide binding, the Ser288 flips down toward the active site (*in*/*down* conformation), establishing a hydrogen bond with the Aspartic acid residue of motif A, involved in the selection of the incoming ribonucleotide (Asp_A_). It is also important to remark that in the previous step, Asp_A_ was hydrogen bonded to a conserved Asn from the motif B (Asn_B_) (Figure 1C,D). Following these changes, new rearrangements occur, including the realignment of the palm motif A, required for catalysis. After the phosphodiester bond formation, the Sholders and Peersen model proposes a movement of the B-loop from “in/down” to “out/down” configuration, resulting in a steric clash between the B-loop and the backbone of the RNA template strand that would facilitate translocation along the RNA and prevent backtracking after translocation. In addition, the finding that the PV 3D^pol^ G289A mutant was able to catalyze single nucleotide addition but was defective for processive elongation provided more evidence of the crucial role of this glycine that confers the flexibility required for the loop movements.

The high sequence and structural conservation of the B-loop among viral polymerases (Figure 3C) strongly suggest that its conformational dynamics would be a common feature of the RNA-dependent RNA polymerases from positive-strand RNA viruses.

### 3.3. Unusual Conformation of Motif A Captured in the Structure of the Cardiovirus EMCV 3D^pol^

The crystal structure of the EMCV 3D^pol^ in its unbound state has been recently solved in two different crystal forms [60]. As expected, the overall architecture of the enzyme was similar to that of the known RdRPs of other members of the *Picornaviridae* family. However, structural comparisons revealed a large reorganization of the active-site cavity in one of the crystal forms. The rearrangement affects mainly the C-terminal loop of motif A, containing the aspartic acid residue involved in incoming rNTP selection (Asp240 in EMCV) (Figure 1B). The heart of this conformational change is that the Asp240 neighbor residue, Phe239, made a drastic movement whereby it is popped out of a hydrophobic pocket in the palm domain to participate in an intriguing set of cation-π interactions in the fingers domain at the edge of the NTP entry tunnel [60]. Another important feature of this altered active site conformation is that the active site of the enzyme was captured in a closed-like state, with the β-sheet supporting motif A totally formed and the catalytic Asp235 positioned in front of the motif C Asp333 (Figure 1B). This active-site conformation has never been observed before in the absence of RNA and a correctly base-paired rNTP. In addition, the N-terminal Gly1 residue was moved out of its binding site, anchored in the fingers domain, toward a totally exposed orientation in the polymerase surface. This is extremely intriguing because like most of the picornaviral RdRPs, the EMCV enzyme is only active when cleaved from the polyprotein to generate an N-terminus with a Gly1 residue [61]. Those observations prompt to hypothesize that this EMCV 3D^pol^ crystal form might represent the structure of the inactive form of the enzyme that would be present in the precursor protein, where Gly1 cannot be buried because is not a terminal residue. However, this hypothesis seems to be in conflict with the structural data currently available for the poliovirus precursor 3CD that also showed Gly1 exposed as part of the flexible linker joining 3C and 3D^pol^ but with the active site in the standard open conformation found in the pre-catalytic complexes [20]. The possible role of the altered conformation of the motif A loop, in particular, of the positioning of the rNTP binding residue Asp240 at the edge of the rNTP entry tunnel, is another mystery to decipher in order to gain insight on the regulation of this enzyme activity.

### 3.4. Conformational Flexibility in the Template Channel

**Figure 4:**
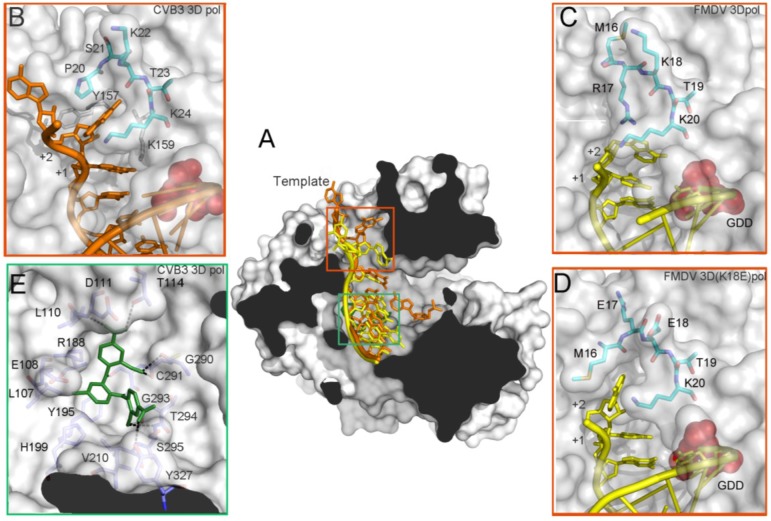
Structure and interactions in the template channel of a picornavirus 3D^pol^. (**A**) The structure of the CVB3 3D^pol^ (PDB id. 4K4Y) has been used as a model, the molecular surface of the polymerase is shown in grey with the acidic residues of the active site in red and the RNA depicted as a cartoon in orange and the FMDV RNA is superimposed in yellow. The non-nucleoside analogue inhibitor is also superimposed in green; (**B**) Structure and interactions in the template channel at the entrance of the active site of CVB3 3D^pol^ (PDB id. 4K4Y), the N-terminal residues 20–24 depicted as sticks in cyan and the RNA in orange and others residues involved in the binding RNA are represented as grey sticks; (**C**) The wild type FMDV 3D^pol^-RNA complex (PDB id. 1WNE); and (**D**) the FMDV 3D^pol^ (K18E)-RNA complex (PDB id. 4WZM); (**E**) Interaction network between GPC-N114 and its binding pocket of CVB3 3D^pol^ represented by surfaces (PDB id. 4Y2A). The polymerase residues in direct contact with the inhibitor are shown with sticks in atom type color with carbon in slate and explicitly labeled. Hydrogen bonds are depicted as dashed lines.

The template channel also exhibits substantial flexibility as visualized by comparing the X-ray structures of different replication elongation complexes [6,7,14,15,19,29,62], as well as predicted by molecular dynamics simulations in a number of picornaviral polymerases [22]. This flexibility appears directly correlated with the role of this channel in driving the template nucleotides toward the catalytic cavity. Of particular importance is the flexible nature of a region included at the 3D^pol^ N-terminus (residues 16 to 20; FMDV numbering), lining the channel that appears to be interacting with the RNA near the single-strand/double-strand junction (Figure 4). The conformational changes occurring in this region would assist the movement of the template nucleotides at the +2 and +3 positions. Interestingly, structural comparisons of the wild type FMDV 3D^pol^ catalytic complexes showed that the basic side chain of Arg17 is involved in different interactions with the template nucleotide t+2 in all complexes analyzed [14,15]. In these complexes, the t+2 nucleotide points towards the active site cavity, stacked with the t+1 nucleotide that is located in the opening of the central cavity, in close contact with the motif B loop (Figure 4B). The equivalent residue of Arg17 in enteroviral polymerases is Pro20 (PV numbering). Structural comparisons of distinct enteroviruses elongation complexes show that Pro20 and its surrounding residues form a conserved pocket where the t+2 nucleotide binds [6,7] (Figure 4). This pocket found in the enterovirus 3D^pol^ seems to be a preformed structure that is also present in the unbound enzymes. In contrast, the FMDV wild type enzyme lacks a preformed pocket in the template channel and, as mentioned above, the t+2 nucleotide is oriented towards the active site cavity (Figure 4), constituting an important structural difference between the enterovirus and FMDV catalytic complexes. Surprisingly, the comparative structural analyses of FMDV 3D^pol^ mutants presenting alterations in RNA binding affinity and incoming nucleotide incorporation, including a remarkable increase or decrease in the incorporation of the nucleoside analog ribavirin, showed important movements in this polymerase region that result in the formation of distinct pockets where the t+2 nucleotide binds [19,62] (Figure 4).

Besides facilitating specific contacts with the RNA template, the dynamic nature of residues lining the template channel should permit the access of the t+1 nucleotide into the 3D catalytic site. The base of the template channel is built mainly by residues of the motif B loop. These B-loop residues are involved in interactions with the t+1 nucleotide in the active site, as well as with the incoming rNTP [15]. Putting together all data is tentative to speculate that the rearrangements in the template channel and the B-loop occur in a concerted manner and that these concerted changes serve to regulate both RNA replication processivity and fidelity.

## 4. Polymerase Oligomerization

Proteins can oligomerize through reversible associations mediated by electrostatic and hydrophobic interactions, hydrogen bonds or by covalent stabilization by disulfide bonds. RdRPs, the enzymes that exclusively belong to the RNA virus world are not the exception. In recent years, the X-ray and Cryo-electron microscopy (cryo-EM) analyses revealed the quaternary structures of a large number of RdRP oligomers, defining the critical residues that lead these associations. Otherwise, complementary biochemical analyses allowed deciphering of the functional roles in most of these arrangements.

RdRP-RdRP interactions to form dimers or higher order oligomers have been predominantly reported for (+) ssRNA viruses, including several Picornavirus [63,64,65,66,67,68], Flavivirus [68,69] and Calicivirus [67,70] enzymes, as well as, in RdRPs of plants [71] and Insect viruses [72]. The homo-interaction of RdRPs was also described in replicases of (−) ssRNA viruses such as influenza A virus [73], and in more distant dsRNA viruses like infectious pancreatic necrosis virus [74]. RdRP oligomerization has been predominantly observed *in vitro* during crystallization, probably produced by the high protein concentration, as well as by other environmental changes like pH or ionic strength. However, intracellular accumulation of oligomeric polymerases was also observed during viral infection of different RNA viruses including PV [64], Sendai virus [75], Rift Valley Fever virus [76] and norovirus [70], among others. These observations suggest that RdRP oligomerization can also be a natural event as a sort of post translational modification. The specificity for the dimerization/multimerization involves distinct surfaces depending on the enzyme, *i.e.*, HCV RdRP dimerization has been proposed to be mediated by the thumb domain [69], whereas, in PV, the polymerase fingers appear to be crucially involved [66]. Contacts between these domains during oligomer formation may cause small conformational changes that are transferred to the active site as an allosteric regulation or could even modify the accessibility of the substrate channels.

**Figure 5:**
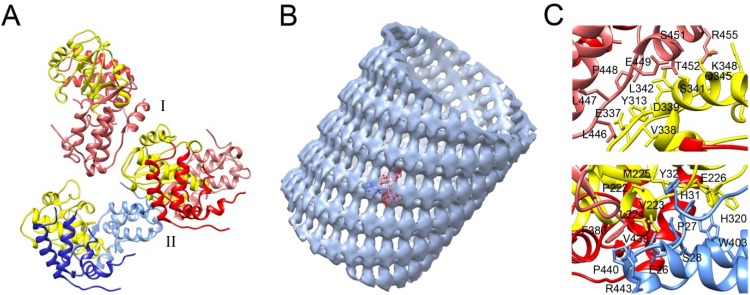
Oligomerization of the PV 3D^pol^. (**A**) Polymerase-polymerase interactions mediated by interfaces I and II, explicitly marked (PDB id. 1RDR) in yellow palm subdomain, in red and blue fingers subdomain and in light colors thumb subdomain; (**B**) Volume map of the reconstructed 3D^pol^ tubes with the crystallographic model positioned inside. The volume map was reproduced from [78] (EM code emd2270); (**C**) Close up of the interface I (upper panel) and interfase II (bottom panel) in the oligomeric tubular array of PV 3D^pol^ according to [78].

The first oligomerization state of an RdRP was described for PV and the nature of the molecular contacts at two different polymerase interfaces, termed I and II, were postulated from the first crystal structure of PV 3D^pol^ [63,64]. The Interface I derived from interactions between the front of the thumb subdomain of one molecule and the back of the palm subdomain of the neighbour molecule in the crystal (Figure 5) whereas interface II involved two N-terminal regions of the polymerase that appeared disordered in this structure [63]. Later on, Lyle *et al.*, using cryo-EM demonstrated that the purified PV 3D^pol^ was able to organize two-dimensional lattices and tubular arrangements formed by polymerase fibres [65] and, recently, the structure of these assemblies has been characterized at the pseudo-atomic level [77,78]. The planar lattices, forming a ribbon-like structure, consist of linear arrays of dimeric RdRPs supported by strong interactions through the interface-I as defined in the PV 3D^pol^ crystal structure [77]. The tubular structure is also formed via interface-I but is also assisted by a second set of interactions placed in interface-II, involving interactions between the fingertips of one molecule and the palm of its contacting neighbour. The fitting of the 3D^pol^ coordinates into the cryo-EM reconstructions showed that interface I connects adjacent dimers by head-to-tail contacts [78] (Figure 5). The relevance of a number of interface II residues in lattice formation was further confirmed by mutagenic analysis. In particular, mutations at residues Tyr32 and Ser438 involved interface II contacts both in planar and tubular array results in a disruption of PV 3D^pol^ lattice formation [77]. Furthermore, several lines of evidence suggested that the PV polymerase can change the conformation upon forming oligomers and, in the tubular assemblies, the porous nature of the polymerase lattice is likely to allow the participation of other viral and cellular proteins [78].

All examples of high-order RdRP assemblies point out that oligomerization may be an advantageous feature providing a functional control, such as allosteric regulation in addition to increasing the stability against degradation and denaturation. The functioning of RdRPs in oligomeric arrays also has an additional advantage, to concentrate the reaction substrates in a physical place and dispose the active sites to use them iteratively.

## 5. Implications for Antiviral Drug Discovery

RdRP synthesize RNA using an RNA template. This biochemical activity, almost exclusive of RNA virures offers the opportunity to identify very selective inhibitors of this viral enzyme. Antiviral drugs targeting the RdRPs may either directly inhibit polymerase activity or essential interactions with the RNA template, or the RdRP-RdRP contacts promoting oligomerization, or interactions with other regulatory proteins. The detailed structural and mechanistic understanding of the conformational changes occurring during catalysis is essential not only for understanding of viral replication at the molecular level but also for the design of novel inhibitors capable of trapping the enzyme in specific conformational states. The Flaviviruses, Hepatitis C virus, Dengue virus and West Nile virus, as well as the calicivirus NV are clear examples of how much effort has been directed towards developing drugs that inhibit viral replication [79,80,81,82]. In general Direct-Acting Antivirals (DAAs), inhibiting RNA replication can be classified into two groups on the basis of their chemical structure and mechanisms of action: Nucleoside Analog (NA) inhibitors and Non-Nucleoside Inhibitors (NNIs). NAs target the active site of the polymerase and need to be converted by the host cell machinery to the corresponding nucleotides, which can either induce premature termination of RNA synthesis [83,84] or be incorporated by the viral polymerase into the nascent RNA, causing accumulation of mutations and contributing to virus extinction through lethal mutagenesis [85].

Conversely, NNIs bind mainly to allosteric pockets of the target polymerase causing alterations in the enzyme dynamics. They might either stabilize an inactive conformation or trap the enzyme in a functional conformation but impeding either the transition between initiation and elongation or the processivity of polymerase elongation [86,87].

Allosteric inhibitors directed against protein-RNA or protein-protein interactions involving viral polymerases are less explored as antiviral drugs. However, effective antiviral molecules that seem to inhibit interactions of the viral polymerase within the replicative complex have been for pestiviruses [88,89] and, a new compound against Dengue virus have been identified that appears to block the RdRP activity through binding to the RNA template channel [20].

In a very recent study, we have identified a novel non-nucleoside inhibitor of 3D^pol^, the compound GPC-N114 (2,2'-[(4-chloro-1,2-phenylene)bis(oxy)]bis(5-nitro-benzonitrile), with broad-spectrum antiviral activity against both enteroviruses and cardioviruses [90]. The X-ray analysis of CVB3 3D^pol^-GPC-N144 co-crystals revealed that the binding site of the compound was located at the junction of the palm and the fingers domains, partially overlapping with the binding site of the templating nucleotide (Figure 4E). The polymerase-inhibitor interactions involved different residues of the conserved motifs G, F, B and A, most found in direct contact with the RNA templates in all picornaviral 3D^pol^-RNA complexes determined so far. Structural comparisons between unbound and GPC-N114 bound CVB3 3D^pol^ revealed that the polymerase did not undergo any major conformational change upon binding of the compound.

Surprisingly, GPC-N114-resistant enterovirus variants could not be obtained, but two EMCV resistance mutations (Met300Val and Ile303Val, in the motif B-loop) were readily selected in the presence of suboptimal concentration of GPC-N114 [90]. The reason for the inability of enteroviruses to develop resistance against GPC-N114 remains to be established. A possible explanation is that mutations that would confer resistance to GPC-N114 also impair binding of the template-primer, thereby preventing replication. Although the exact reason remains to be determined, the structural data suggest that, in contrast to most allosteric binding sites, the GPC-N114-binding cavity in enterovirus 3D^pol^ lacks the conformational plasticity required to develop resistance. In contrast, EMCV 3D^pol^ appears to be sufficiently plastic to allow for compound-resistance substitutions. As expected, structural comparisons showed high similarity between the CVB3 and the EMCV enzymes, but a major difference existed in the main interactions established between these polymerases with the inhibitor that could possibly underlie the differences observed in the emergence of resistant mutations. A key interaction of the CVB3 3D^pol^-GPC-N114 was mediated by Tyr195 (Figure 4E). In contrast, the equivalent residue in EMCV 3D^pol^ is Ala, resulting in a weaker interaction with the compound. This weaker interaction together with an increment of the flexibility in the compound-binding area, induced by the B loop resistance mutations, might result in a decrease of GPC-N114 binding to the EMCV enzyme.

In summary, the identification of this novel drug-binding pocket in the picornaviral 3D^pol^ might serve as a starting point for the design of new antiviral compounds targeting the template-binding channel.

## 6. Conclusions

RNA-dependent RNA polymerases (RdRPs) play central roles in both transcription and viral genome replication. In picornaviruses, these functions are catalyzed by the virally encoded RdRP, termed 3D^pol^. 3D^pol^ also catalyzes the covalent binding of two UMP molecules to a tyrosine on the small protein VPg. Uridylylated VPg then serves as a protein primer for the initiation of RNA synthesis.

The ever growing availability of structures of picornaviral catalytic complexes provided an increasingly accurate picture of the functional steps and regulation events underliying viral RNA genome replication. Data currently available provides high-resolution pictures for a range of conformational states associated to template and primer recognition, VPg uridylylation, rNTP recognition and binding, catalysis and chain translocation. Such structural information is providing new insights into the fidelity of RNA replication, and for the design of antiviral compounds.

Protein primed mechanism of replication initiation mediated by VPg appears to be a process that involves more than one VPg binding site in 3D^pol^ possibly at different stages of the virus replication initiation process. Although the number of 3D^pol^-VPg structures available show individual snapshots of the process, to obtain a global picture of the assembly, regulation and dynamics of complete replication initiation complexes, requires further analyses of high order assemblies formed not only by the polymerase and VPg but also involving different viral and host proteins, protein precursors and RNA templates. Such structures should provide a more detailed view of the molecular events underlying the initiation of picornavirus genome replication.

The structures of a large number of picornavirus replication-elongation complexes captured subtle conformational changes associated with nucleotide selection and active site closure. Among these movements, the motif B-loop assists in the positioning of the template nucleotide in the active site. Binding of the correct nucleotide then induces the β-strands realignment in the palm subdomain and repositioning of the motif A aspartate for catalysis. Steric clashes between the motif B-loop and the template RNA would finally promote translocation.
